# Considerations about Cytotoxicity of Resin-Based Composite Dental Materials: A Systematic Review

**DOI:** 10.3390/ijms25010152

**Published:** 2023-12-21

**Authors:** Kacper Wiertelak-Makała, Izabela Szymczak-Pajor, Kinga Bociong, Agnieszka Śliwińska

**Affiliations:** 1 Student Scientific Society of Civilization Diseases, Medical University of Lodz, 251 Pomorska Str., 92-213 Lodz, Poland; 2 Department of Nucleic Acid Biochemistry, Medical University of Lodz, 251 Pomorska Str., 92-213 Lodz, Poland; 3 Department of General Dentistry, Medical University of Lodz, 251 Pomorska Str., 92-213 Lodz, Poland

**Keywords:** dental materials, resin-based composite, biocompatibility, cytotoxicity, oxidative stress, apoptosis

## Abstract

The dental material industry is rapidly developing resin-based composites (RBCs), which find widespread use in a variety of clinical settings. As such, their biocompatibility has gained increasing interest. This literature review presents a summary of research into the cytotoxicity of methacrylate-based composites published from 2017 to 2023. Subject to analysis were 14 in vitro studies on human and murine cell lines. Cytotoxicity in the included studies was measured via MTT assay, LDH assay, and WST-1 assay. The QUIN Risk of Bias Tool was performed to validate the included studies. Included studies (based entirely on the results of in vitro studies) provide evidence of dose- and time-dependent cytotoxicity of dental resin-based composites. Oxidative stress and the depletion of cellular glutathione (GSH) were suggested as reasons for cytotoxicity. Induction of apoptosis by RBCs was indicated. While composites remain the golden standard of dental restorative materials, their potential cytotoxicity cannot be ignored due to direct long-term exposure. Further in vitro investigations and clinical trials are required to understand the molecular mechanism of cytotoxicity and produce novel materials with improved safety profiles.

## 1. Introduction

### 1.1. Background

Dental caries, the most common oral health disorder, is a societal disease that affects patients of every age and social group worldwide [1,2]. Its multifactorial mechanism is explained by the loss of minerals from susceptible enamel, cementum, and dentin due to an acidic environment induced by cariogenic microorganisms when physiological remineralization processes are insufficient to restore lost minerals [1,2]. Cariogenic bacteria play an important role in the development of caries. As such, it can be defined as an infectious, dental plaque-dependent and biofilm-mediated disease [1,2]. Unbalanced mineral loss disrupts the structure of enamel, cementum, and dentin and leads to the development of carious lesions, which seriously impact masticatory function, cause pain, and have a negative aesthetic impact [3]. As such, carious lesions require clinical intervention: the removal of carious tissue and its replacement with either a direct filling or, in extensive tissue loss, prosthetic restoration [1,2]. Hard tissue loss caused by other factors, such as trauma, requires a similar restoration process [3].

Methacrylate-based composite materials have been used as filling materials in dentistry since the 1960s [4]. Since then, there have been many developments in the field of dental material science, resulting in the production of a new class of materials with versatile applications. Nowadays, resin-based composite restorative materials (RBCs) are widespread as the first choice of restorative material, largely replacing amalgam as the gold standard in conservative dentistry [5,6,7]. This change has been further accelerated by the Minamata Convention on Mercury, which advised a phase-down in the application of dental amalgam due to environmental concerns regarding the impact of mercury pollution [3,8]. The interdisciplinary interactions of dentistry, material science, and molecular biology have led to dynamic and productive developments in the field in recent years. The paradigm shift to minimally invasive dentistry in clinical practice and the increasing demand for aesthetic, tooth-colored restorative materials contributed to the popularity of RBCs [5,6,8,9,10,11,12,13]. The major advantages of RBCs as the gold standard of restorative material are that they are adhesive to the tooth structure, restore the structural integrity of the tooth, and have excellent aesthetics as well as clinically favorable parameters of mechanical performance, durability, and resistance to degradation [3,6,8,13].

Currently, RBCs are rapidly developing, with 3D-printable resins or bioactive materials with antibacterial and remineralizing properties being tested and introduced into the dental market [1,2,13,14,15,16]. Novel composites seek to not merely replace but fully replicate lost tissues [17]. Challenges such as toxicity, high polymerization shrinkage (and, in consequence, contraction stress), secondary carries, wear resistance, chipping and bulk fracture, and degradation in time of resin composite-based restorations are still being explored [3,6,7,18,19,20]. The degradation of adhesive materials and their bonds with the dentin is a complex, multifactorial process resulting from the hydrolysis of the resin as well as masticatory and hydraulic stress [21]. Collagen fiber hydrolysis and the enzymatic activity of host-derived matrix metalloproteinases and cysteine cathepsins further contribute to the degradation of the hybrid layer interface of restorative material and tooth structure [21]. Resin-based composites are applied in combination with adhesive systems following a wide range of clinical protocols, such as two-step etch and rinse, three-step etch and rinse, one-step self-etch, two-step self-etch, and universal adhesives, further modifying the bond strength of composite restorations [20]. These factors allow for degraded or unpolymerized monomers to diffuse through saliva and the dentinal tubules and interact directly with the pulp and soft tissues of the oral cavity, leading to biological interactions described in the further parts of this manuscript. These challenges are being addressed via the modification of RBC composition in manufacturing and new clinical application protocols.

The objective of this review was to evaluate the biocompatibility of dental resin-based composite materials and their mechanism of toxicity in in vitro models. The following question was formed to guide the research: “In what way do resin-based composite materials used in dentistry induce cytotoxicity on the cells of oral cavity tissues”?

### 1.2. Basic Characteristics of Resin-Based Composites

Resins are a wide class of viscous materials capable of hardening. They are required to meet appropriate criteria in order to be used in dental restorations. Their mechanical properties, wear resistance, and optical properties are expected to be similar to dental tissue [22]. They should present a high color stability. Dental materials should be adhesive to the tissues and easy to work with [22].

Since resin-based composites are intended to replace carious tissues, they must possess mechanical properties required to withstand stress without damage. Compressive strength, bending strength and elastic modulus are the main experimental criteria tested while selecting composite materials [22].

Resin-based composites include a diverse group of complex polymers [23]. Their change from viscous liquid to hardened solid state is caused by a polymerization reaction triggered by external energy in the form of heat, chemical, or radiant energy conditional on the presence of initiators [5]. Most monomers used in RBCs are linear molecules with a methacrylate group at each end [10]. Their conversion to complex polymers occurs via vinyl-free radical chain growth polymerization divided into three stages: initiation, propagation, and termination [5,10]. There are two modes of initiation: chemically activated and light-activated [3,19]. Activated photo-initiators generate free radicals, which convert double C=C bonds of methacrylate monomers into single C-C bonds, triggering a chain reaction. It should be noted that polymerization is never fully complete, and the maximum conversion of monomers in the cured composite is measured as the Degree of Conversion of resin [3,4,10,11,15,24]. Reduction in the filler–matrix ratio, as well as using nano-filled composites rather than particle-filled materials, tend to increase the Degree of Conversion [22]. Curing depth is another important consideration. The time of irradiation, effective wavelength, and light intensity of the curing lamp, and its distance from the material’s surface all influence the curing depth [22]. Due to incomplete conversion, as well as aging, mechanical weardown, hydrolysis, and enzymatic degradation over time, the elution of uncured, leachable monomers—as well as initiators and other additives—into the liquids of the oral cavity occurs [3,4,7,11,12,24,25,26].

**Figure 1:**
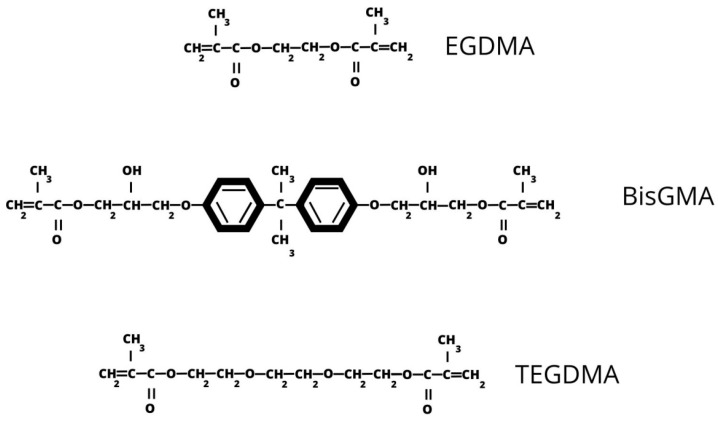
Most common resin monomers: EGDMA—ethylene glycol dimethacrylate; BisGMA—bisphenol A-glycidyl methacrylate; TEGDMA—triethylene glycol dimethacrylate.

RBCs consist of an organic resin matrix (typically 15–50% of its weight), inorganic filler particles (reinforcing phase), a coupling agent, and a photo-initiator [5,8,19,22,27]. The chemical composition and function of each phase are summarized in Table 1. Most common resin monomers chemical structure is presented in Figure 1.

### 1.3. Biological Properties of Resin-Based Composites

Biocompatibility is a vital characteristic of biomaterials, defining organisms’ reactions to them [29]. It can be defined as the ability of a biomaterial to produce a suitable host reaction when applied according to standard procedure [12,25]. To be classified as biocompatible, biomaterials must avoid toxic, harmful, and otherwise physiologically undesirable reactions, such as cytotoxicity, induction of oxidative stress, induction of inflammatory response, mutagenicity, and immunological rejection [24,25,30,31]. Biocompatible materials should also not interfere with the healing process [30]. Toxicity is defined as the damage induced by the biomaterial to the organism [12]. Mercury toxicity controversy has been a major rationale behind the replacement of amalgam with RBCs as the golden standard of restorative material in dentistry [5]. A comprehensive understanding of the biocompatibility and potential toxicity of RBCs is of vital importance since dental biomaterials are in long-term direct and indirect contact with the surrounding tissues of the oral cavity. As such, ideal biomaterials should be chemically stable, biocompatible, and tasteless [11,12,22,25]. In the oral cavity, RBCs are exposed to saliva, food, bacteria, and by-products of their metabolism, changes in pH and temperature, as well as mechanical weardown [12]. In commercially available RBCs, there is some extent of degradation and solution leading to the elution of toxic components, making local exposure via both direct contact of dentin, pulp, and gingival cells as well as indirect exposure of gingiva via saliva possible and worth consideration [4,12,25,32]. Concerns have been raised regarding the allergies and hypersensitivities in patients as well as cytotoxic, mutagenic, and estrogenic effects on cells exposed to residual monomers [4]. Biocompatibility is important not only for the patients but also for the medical professionals [30]. Adverse skin and mucosal reactions have been reported in about 12% of patients and 27% of dentists utilizing RBCs [12,25]. Resin is responsible for such adverse reactions, which are mostly induced by uncured RBCs.

So far, dental materials were intended to simply replicate missing tissues. However, in recent years, developments have been made to introduce a new generation of bioactive materials. Remineralizing and antibacterial properties or the ability to inhibit biofilm formation or neutralize acids are researched [33].

Cytotoxicity, the main point of focus of this manuscript, can be defined as toxicity caused by exogenous substances in living cells. Uncured composites exert much greater cytotoxicity due to the higher content of free monomers able to induce oxidative stress [29]. The consideration of cytotoxicity is of paramount importance, as resin-based composites are in prolonged close contact with the pulp and gingival.

## 2. Methods

### 2.1. Research Strategy

A PICO framework was used to guide the literature search. The following question, as mentioned above, was posed: “In what way do resin-based composite materials used in dental practice induce cytotoxicity on the oral cavity tissues”? The target population (P) was human and animal cells naturally found in the oral cavity tissues (studied in vitro), and the intervention (I) was exposure to commercially available resin-based materials (adhesive systems, composite resins, and resin luting agents) or to experimental resins or to free resin monomers, the comparison (C) was untreated cells or positive controls, and the outcome (O) was a change in the viability of the treated cells.

In order to research the current literature, the library of the Medical University of Lodz, Google Scholar, and PUBMED search engines were used to identify relevant papers. Subject to screening were original studies: in vitro studies. The search for literature was performed up to 25 May 2023. The following keyword combinations were employed: “cytotoxicity of dental materials” OR “cytotoxicity of resin composite” OR “biocompatibility of dental materials” OR “biocompatibility of resin composite” OR “oxidative stress of dental materials” OR “oxidative stress of resin composite” OR “genotoxicity of dental materials” OR “genotoxicity of resin composite” OR “mutagenicity of dental materials” OR “mutagenicity of resin composite”. Only English language literature was analyzed.

### 2.2. Study Selection

Abstracts of publications returned from the search engines were selected using inclusion and exclusion criteria summarized in Table 2. Duplicates were removed manually. A hand search was performed in the reference lists of valuable studies to identify additional relevant papers. For all publications that met the inclusion criteria, the electronic version of the paper was retrieved in full and analyzed.

### 2.3. Risk of Bias

The quality of selected in vitro studies required further validation. The Quality Assessment Tool for In Vitro Studies (the QUIN Tool) developed by Sheth et al. was used in the process [34]. The QUIN Tool takes into consideration a list of 12 criteria: clearly stated aims/objectives; detailed explanation of sample size calculation; detailed explanation of sampling technique; details of the comparison group; detailed explanation of methodology; operator details; randomization; method of measurement of outcome; outcome assessor details; blinding; statistical analysis; and presentation of results. Each criterion is then assigned a score: score = 2 for “adequately specified”, score = 1 for “inadequately specified”, or score = 0 for “not specified”. Criteria that are recognized as not applicable are not included in the final score. The final score is obtained using the following formula:

$$Final\;score=\frac{Total\;score\times 100}{2\times number\;of\;applicable\;criteria}$$

The obtained scores are used to assign the risk of bias: >70% for low risk of bias, 50–70% for medium risk of bias, and <50% for high risk of bias. Studies assigned a high risk of bias were rejected.

## 3. Results

### 3.1. Study Selection

**Figure 2:**
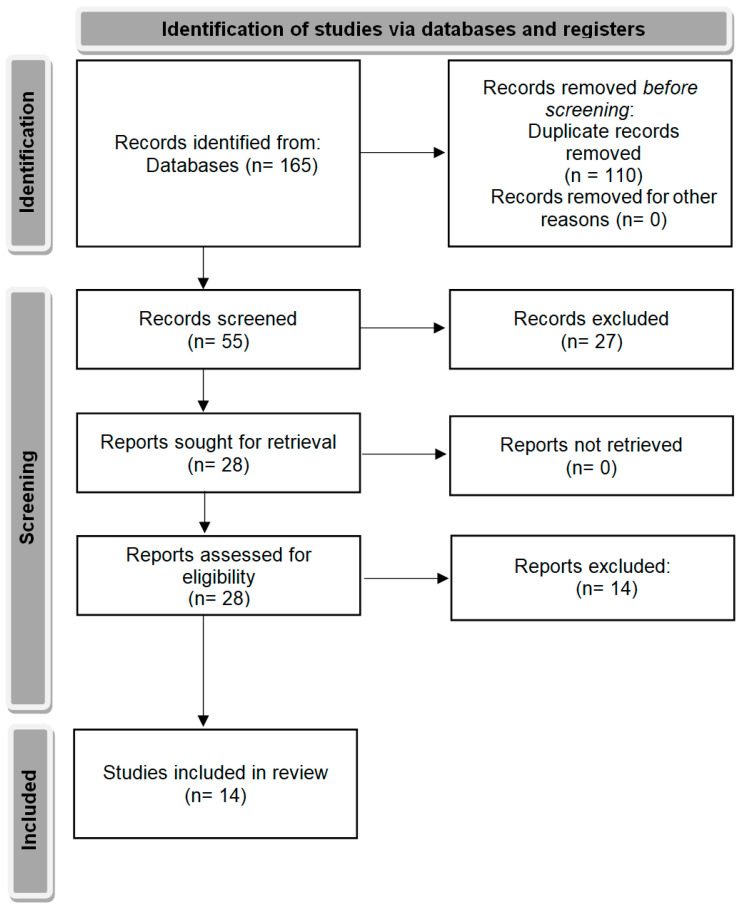
PRISMA flow chart of the study selection process.

There were 55 studies identified in the databases. Of these studies, 27 were excluded from screening. The remaining 28 papers were retrieved and assessed for eligibility. Of these, 14 studies were selected for further analysis based on the inclusion and exclusion criteria presented in Table 2. PRISMA flow chart of the study selection process is presented in Figure 2 [35]. A brief summary of the included studies is presented in Table 3.

### 3.2. Risk of Bias

The risk of bias for selected studies was assessed using the Quality Assessment Tool for In Vitro Studies (the QUIN Tool). Out of 14 studies, there were 9 that were assigned a low risk of bias and 5 with a medium risk of bias.

### 3.3. Cytotoxicity Results

It should be noted that studies included in this review had differing methodologies. One major difference was the material of choice in the study. For the purpose of this review, included studies were divided with regard to the method of exposure. Group one analyzed free resin monomers suspended in a medium. Group two focused on eluates obtained from commercially available composites after polymerization.

Four types of cell viability assays were used to assess the cytotoxicity of tested materials: MTT, XTT, WST-1, and LDH. MTT assay is a colorimetric assay that utilizes 3-(4,5-dimethylthiazol-2-yl)-2,5-diphenyl-2H-tetrazolium bromide, a monotetrazolium salt. Its reduction results in the formation of a violet water-insoluble formazan [52]. XTT assay is a related procedure that uses as its reagent 2,3-bis (2-methoxy-4-nitro-5-sulfophenyl)-5-[(phenylamino) carbonyl]-2H-tetrazolium hydroxide [53]. WST-1 is another tetrazolium salt assay that utilizes 2-(4-iodophenyl)-3-(4-nitrophenyl)-5-(2,4-disulfophenyl)-2H-tetrazolium [54]. LDH assay is based on the activity of L-lactate dehydrogenase [55]. All assays are commonly used in testing cytotoxicity and can be treated as the gold standard.

Neves et al. revealed in MTT assay that BisGMA, UDMA, and TEGDMA induced dose-dependent cytotoxicity on human peripheral blood mononuclear cells with toxic effects present at high concentrations: BisGMA at 0.06–1 mM induced a 44–95% decrease in mitochondrial activity, UDMA at 0.05–2 mM caused 50–93% decrease, and TEGDMA at 2.5–10 mM induced a 26–93% decrease [36]. Schneider et al. took on a different approach, measuring the LDH release of human dental pulp cells as an indicator of toxicity of their exposure to dental composite resin monomers: BisGMA, UDMA, and TEGDMA [42]. Their findings indicated BisGMA and UDMA as highly toxic, with BisGMA-induced toxicity beginning at the concentration of 30 μm and UDMA starting at 100 μm. TEGDMA failed to cause toxicity at any concentration tested [42]. Lovász et al. used a water-soluble tetrazolium salts (WST-1) colorimetric assay to measure the viability of human dental pulp cells exposed to TEGDMA monomers [43]. They noted a significant reduction in viability after exposure to 1.5 and 3 mM of TEGDMA for 24 h. Lower concentrations at this time did not produce significant effects [43]. In another study by Lovász et al., human dental pulp cells exposed to 0.75, 1.5, and 3 mM TEGDMA for 5 days were analyzed via WST-1 assay [45]. After 24 h, there was a decrease in viability from 1.237 WST values in the control group to 0.970, 0.814, and 0.518 WST values for 0.75, 1.5, and 3 mM, respectively. Second-day results presented a decrease from 1.961 to 1.290, 0.472, and 0.056 WST values in the same group order. Similarly, the fifth-day results were a decrease from 2.259 to 0.893, 0.105, and 0.089 WST values [45]. Sun et al. studied HEMA cytotoxicity on human dental pulp cells incubated for 72 h with 50, 100, 200, 400, 800, 1000, 1500, 2000, 2500, and 3000 μg/mL HEMA [46]. Cell viability levels were consistently reduced. Yang et al. investigated the cytotoxicity of TEGDMA monomers on murine macrophages via MTT assay [47]. The cells were incubated with or without TEGDMA for 24 h. TEGDMA was found to induce approximately 75% cytotoxicity in comparison to the control group [47]. Lee et al. conducted a similar study on murine macrophages incubated with 0, 0.5, 1, 5, and 10 mM HEMA for 24 h, testing the cytotoxicity with an MTT assay [48]. The results presented a dose-dependent cytotoxicity, with a decrease in viability by almost 20% for 1 mM, 40% for 5 mM, and 55% for 10 mM [48]. Chang et al. studied murine macrophages incubated with UDMA at 0, 0.1, 1, 10, and 100 μM for 24 h, measuring the cytotoxicity via LDH (lactate dehydrogenase) assay [49]. They observed 12% cytotoxicity for 1 μM and as high as 40% cytotoxicity for 10 μM [49].

Cengiz et al. recorded a reduction in MTT absorbance in murine fibroblasts exposed to Signum and Adoro composites over 2 weeks, with significant differences for both materials at each incubation time [37]. Carrillo-Cotto analyzed MTT assay in human spontaneously transformed aneuploid immortal keratinocytes exposed to adhesive systems OptiBond FL, Clearfil SE Bond and Adper Single Bond Universal, conventional composite resin Filtek Z350 XT, flowable composite resin Filtek Flow Z350 XT, self-adhesive composite resin Dyad Flow and luting agents Variolink II and RelyXU200 in combinations simulating their clinical use with or without the presence of dentin [38]. They observed that cytotoxicity varied with the combination of materials tested and that for all tested materials, most toxic effects were observed in the first 24 h. Dentin was found to increase cell viability. Beltrami et al. used an MTT assay to determine the cytotoxicity of immortalized human gingival fibroblasts exposed to different nano-hybrid composite resins [39]. It was found that after 72 h of incubation, the cell viability was significantly lower than after 48 h of incubation for all tested materials except for Enamel Plus HRi and G-aenial. After 48 h, Omnichroma, Omnichroma Blocker, Admira Fusion x-tra, and Enamel Plus HRi Bio Function Enamel showed the lowest grade of cytotoxicity, with cell viability above 80% [39]. Enamel Plus HRi and G-aenial showed, respectively, severe and moderate toxicity after 48 h. After 72 h of incubation, Omnichroma and Omnichroma Blocker showed mild cytotoxicity with a significant decrease in cell viability rates as compared to levels after 48 h. Admira Fusion x-tra and Enamel Plus HRi Bio Function Enamel showed a significant reduction to moderate cytotoxicity after 72 h. G-aenial Flo X and Enamel Plus HRi Bio Function Bio Dentine showed similar results after 48 h and 72 h. Both materials showed a lower cell viability rate after 72 h as compared to 48 h incubation [39]. Kavuncu et al. tested three composite materials, Admira Fusion, Charisma Topaz, and Estelite Quick Sigma, on human gingival fibroblasts and periodontal ligament fibroblasts [40]. In comparison to the control group, Admira Fusion, and Estelite Quick Sigma presented no cytotoxic effects on gingival fibroblasts after 24 h incubation. Only Charisma Topaz was cytotoxic to gingival fibroblasts at this time. After 1 week of incubation, the viability of gingival fibroblasts exposed to Charisma Topaz was significantly lower than the control, while the other materials were similar to the control. In contrast, Charisma Topaz did not produce significant cytotoxicity on periodontal ligament fibroblasts after 24 h of incubation and was the only cytotoxic material in 1 week of exposure [40]. Sulek et al. tested the cytotoxicity of Charisma, Estelite Sigma Quick, and Filtek Z250 on human gingival fibroblasts via MTT assay [41]. All tested materials significantly decreased cell viability while freshly cured. Charisma generated a 58% decrease in cell viability, and Filtek Z250 and Estelite Sigma Quick resulted in a 31% and 22% decrease, respectively. In delayed toxicity testing by LDH assay, pre-incubated Charisma toxicity was significantly lower, Estelite Sigma Quick resulted in similar toxicity, and Filtek Z250 was significantly more toxic [41]. Agnes et al. examined human dental pulp cells exposed to Flow Line and Durafill resin composites combined with mineral trioxide aggregate (MTA) and Dycal capping materials using LDH assay [44]. Their findings showed that 24 h exposure to Durafill and Flow Line resulted in cell death rates of approximately 35–40% and 35%, respectively. It should be noted that exposure to the combination of Durafill with Dycal did not alter toxicity significantly, and Flow Line with Dycal actually decreased cell death rates to around 15%. Meanwhile, the combination of MTA with Durafill enhanced Durafill cytotoxicity up to approximately 85%, and the cytotoxicity of Flow Line did not alter significantly [44]. Wawrzynkiewicz et al. used XTT assay (2H-tetrazolium-5-Carboxanilide colorimetric assay) to assess the cytotoxicity of dental adhesives—All-Bond Universal, CLEARFIL Universal Bond Quick, G-Premio BOND and Single Bond Universal—on human monocytes/macrophage peripheral blood cells [50]. Their results indicated significant differences in the cytotoxicity of tested eluates. Only G-Premio BOND was found to significantly decrease the cell viability by around 22% in comparison with the control. In another study by Wawrzynkiewicz et al. on human monocytes/macrophage peripheral blood cells, only OptiBond Universal induced a significant decrease in cell viability by around 25% [51].

The results presented above indicate a dose- and time-dependent cytotoxicity of resin-based composite. As could be predicted, increasing the dose of the material and time of exposure enhance cytotoxicity. It should be noted, however, that this review is based entirely on the results of in vitro studies. As such, it is up for further consideration to what extent the conditions of included studies are representative of the actual environment of the oral cavity. The resin monomer concentration is likely to be lower in vivo. Additionally, concentration fluctuates throughout the day due to many changing factors in the oral cavity. These arguments should be well considered while interpreting the cytotoxicity results of in vitro studies. Moreover, the type and origin of cells used in the included studies should be considered while drawing comparisons. These considerations will be further reflected upon in Section 4 (Discussion) of this review.

### 3.4. Summary of Study Characteristics

A brief summary of the included studies is presented in Table 4.

## 4. Discussion

### 4.1. Mechanism of Toxicity

Resin-based materials are in prolonged, intimate contact with the oral cavity. This includes both direct contact between the filling and restored dentin, enamel, or adjacent gingiva, as well as indirect contact with the dentin and pulp via dentinal tubules or soft tissues via saliva. As such, cytotoxicity should be thoroughly studied and its mechanism well understood so that novel materials with improved safety profile can be developed.

Incomplete polymerization plays a major role in RBCs cytotoxicity, allowing for the elution of toxic methacrylate and dimethacrylate monomers: BisGMA, TEGDMA, UDMA, HEMA, BisEMA [11,15,41]. Most authors agree that the extent of the toxic effect is dependent on several factors, e.g., the chemical composition of the material, dose, and time of exposure, as well as internal factors, e.g., cell line, cell lining parameters, cellular membrane integrity, cell volume, and cytoplasm volume, the refractive index of the cell, the propensity to cleave DNA, and related nuclear condensation [12,36,37,38,39,40,43,45,46].

Smaller monomers with hydrophilic properties (e.g., HEMA and TEGDMA) have been found to penetrate cellular membranes, inhibit glutathione and lipids synthesis, and induce damage to mitochondria and DNA [12,25,38,42,45]. This damage to DNA is best understood as the loss of integrity and fragmentation of DNA strands [48]. Mitochondrial dysfunction can be explained by the generation of reactive oxygen species and depolarization of mitochondria, which leads to caspases-mediated apoptosis [48,49]. Mitochondria play a vital role in cellular metabolism and homeostasis, and their dysfunction initiates cell death [49]. Toxicity of BisGMA can be connected to this monomer being a derivative of Bisphenol A (BPA), which is known to be involved with genital developmental problems, immune function, thyroid function, and neurodevelopment in children [15,25]. Synthetic pathways of BisGMA production that do not require the use of bisphenol A have been introduced, but BPA presence has been found in urine and saliva of patients after dental treatment [19].

Monomers such as TEGDMA have been found to increase the generation of mitogen-activated protein kinases (MAPKs), which may interfere with biomineralization and initiate apoptosis [12,47].

Suggested molecular mechanisms of toxicity after exposure to RBCs include the depletion of glutathione (GSH), a tripeptide responsible for maintaining the redox balance of the cell [12,25,30,38]. GSH can be consumed to detoxify the monomers even in non-lethal concentrations, which induce little changes in viability [30]. Schneider et al. tested the effects of BisGMA, UDMA, and TEGDMA monomers on cystine uptake in dental pulp cells since it has been demonstrated to alter cellular glutathione levels [42]. After 2 h, BisGMA and UDMA decreased cystine uptake, and TEGDMA increased it, with significant changes observed at the monomer concentrations of 300 μM [42]. After 48 h, a significant decrease in cystine uptake was observed for BisGMA and UDMA at concentrations of above 30 μM and 100 μM, respectively. Interestingly, lower UDMA concentrations caused increased uptake at 48 h. TEGDMA induced increased cystine uptake [42]. An incoherent relation between cystine uptake and cellular GSH level was observed, contrary to the expectation that since cystine is necessary for GSH production, its changes would mirror one another. At 2 h, there was a noticeable decrease in cellular GSH for all monomers independently of the concentration. At 48 h, there was a dramatic decrease in cellular GSH levels for BisGMA and UDMA in concentrations of 30 μM and above. At the same time, TEGDMA caused a slight increase at 100 μM and a decrease at 300 μM [42]. Depletion of GSH levels upsets the redox balance of the cell. Reactive oxygen species are produced in increased quantities, inducing redox imbalance resulting in damage to DNA and cell death [12,30]. This redox imbalance activates adaptive cell mechanisms, up- and down-regulating enzyme systems [30].

Lovász et al. found via Western blotting that TEGDMA exposure induced strongly increased levels of metalloproteinases MMP-2, MMP-8, and MMP-9 in dental pulp cells at the concentration of 0.2 mM. These metalloproteinases are protein hydrolases produced in the pulp and found in dentin. They can be divided into two groups. MMP-2 and MMP-9 are classified as gelatinases involved in tissue remodeling and tertiary dentin formation [43]. MMP-8 is classified as a collagenase involved in the organization of the pre-mineralized collagen fiber network in the dentin [43]. A lower concentration of 0.1 mM increased MMP-2 expression alone, and a higher 0.75 mM concentration increased MMP-2 significantly and MMP-8 slightly [43]. HEMA has been found to inhibit MMP-2 and MMP-9 expression by Sun et al. [46].

Sulek et al. analyzed changes to the expression of two epigenetic and biochemical biomarkers of toxicity: miR-9 and heat shock protein 70 (HSP70) in human gingival fibroblasts. mi-R9 is a stress-related micro-RNA with indicative potential for the transition of healthy fibroblasts into cancer cells. This micro-RNA binds to 3′-untranslated regions of the target mRNA, regulating the transcription of several genes relevant to cell physiology and pathology, and its increased level is typical for epithelial cells undergoing epithelial-mesenchymal transition, indicating cancer transformation [41]. HSP70 is an important chaperone and stress marker protein involved in protection mechanisms against thermal, chemical, and oxidative stress, responsible for refolding damaged proteins and inhibiting apoptosis [41]. Sulekat et al. observed that miR-9 was significantly increased in all groups—there was a 3.4-fold increase in cells incubated with Filtek Z550 and less than that 2-fold increase for Charisma and Estelite Quick Sigma [41]. As for HSP70, only Charisma induced a significant 7-fold increase in its expression [41]. In other studies, HEMA and TEGDMA were observed to decrease heat shock protein expression in human monocytes [25].

The production of reactive oxygen species is another important consideration [25,45]. While reactive oxygen species are physiologically instrumental in immunoregulation, and antimicrobial and antiviral functions, their excessive production may lead to cellular damage, DNA damage, lipid peroxidation, and inflammatory response [47,48]. Since all RBCs contain toxic monomers and free-radical-based photo-initiators, they can be expected to produce oxidative stress. As mentioned above, depletion of glutathione levels upsets the redox balance of the cell, and reactive oxygen species are produced in increased quantities, inducing redox imbalance [12,30]. Sulek et al. analyzed oxidative stress in human gingival fibroblast cells grown for 3 and 6 h with Charisma, Estelite Quick Sigma, and Filtek Z550. In all samples, there was a significant increase in the mean DCF fluorescence [41]. Filtek Z550 induced the highest increase at 12-fold. A slightly lower 10-fold and 9-fold increase was observed in fluorescence intensity for Charisma and Estelite Quick Sigma, respectively [41]. Schneider et al. demonstrated a significant increase in free radicals in high BisGMA and UDMA monomer concentrations, while TEGDMA had no influence on free radicals in dental pulp cells [42]. The accumulation of reactive oxygen species induces oxidative stress, damaging DNA, lipids, and proteins. Genotoxicity and induction of apoptosis by accumulation of reactive oxygen species are strictly associated with cytotoxicity.

Some authors have demonstrated that RBCs influence inflammatory response in soft tissues adjacent to the restoration [39]. Incubation of macrophages with TEGDMA initiates several proinflammatory mechanisms, such as the upregulation of cyclooxygenase-2 and inducible nitric oxide synthase [47]. Neves et al. investigated the influence of BisGMA, TEGDMA, and UDMA on pro-inflammatory cytokine IL-1β and TNF-α release on peripheral blood mononuclear cells incubated with TC_20_ concentration of those monomers [36]. Those cells typically exhibit an increased cytokine production when exposed to *Porphyromonas gingivalis*. BisGMA did not influence the expression of cytokines in any way. However, TEGDMA had an inhibitory influence on the secretion of both IL-1β and TNF-α, and UDMA decreased IL-1β alone in cells exposed to *P. gingivalis* [36]. These findings suggest that monomers could interfere with the local immune inflammatory response. However, no negative impact was observed in cells that were not stimulated with *P. gingivalis*, suggesting that low concentrations of resin monomers assure relative safety [36]. It should be noted that the outcomes could be influenced by a decreased number of viable cells capable of cytokine production.

In animal studies, methacrylates increased the number of micro-nucleated cells in bone marrow, indicating mutagenicity [25]. Sulek et al. found that Charisma produced a 5-fold increase in hypodiploid cell numbers in comparison with the control group, signifying high DNA damage in human gingival fibroblasts [41]. In the same study, Estelite Sigma Quick and Filtek Z550 induced a 2- and 3-fold increase in damaged cells, respectively [41].

### 4.2. Impact on Cell Viability

Most studies conclude that RBCs induce cytotoxicity in in vitro cell models and that the impact on cell viability is dose- and time-dependent and changes depending on the chemical composition of the product and is specific for each cell line [36,37,38,40,49].

Neves et al. found that three tested monomers—BisGMA, UDMA, and TEGDMA—caused dose-dependent cytotoxicity on peripheral blood mononuclear cells, as evidenced by inhibited mitochondrial metabolic activity [36]. They established the following order of toxicity, based on TC_50_ (concentration that caused a 50% decrease in cell viability) and TC_20_ (concentration that caused a 20% decrease in cell viability) values: BisGMA > UDMA > TEGDMA with TC_50_ values of 69.0 mM, 505.0 mM, and 3161.0 mM; and TC_20_ values of 50.5 μM, 167.0 μM, and 2150.0 μM, respectively [36]. Schneider et al. established that BisGMA and UDMA caused cytotoxicity in concentrations beginning at 30 μm and 100 μm, respectively [42]. Most studies support that the order of cytotoxicity of monomers is BisGMA > UDMA > TEGDMA > HEMA [41,42].

Beltrami et al. investigated the biocompatibility of several RBCs on human gingival fibroblasts. MTT assay revealed that after 72 h of incubation, cell viability was significantly lower than after 48 h of incubation for all tested materials except for Enamel Plus HRi and G-aenial [39]. Both Enamel Plus HRi and G-aenial did, however, show severe and moderate cytotoxicity after 48 h, respectively. After 48 h, Omnichroma, Omnichroma Blocker, Admira Fusion x-tra, and Enamel Plus HRi Bio Function Enamel presented a low degree of cytotoxicity, with cell viability rates above 80% [39]. After 72 h, Omnichroma and Omnichroma Blocker induced mild cytotoxicity, with a significant decrease in cell viability rates in comparison to 48 h incubation [39]. Admira Fusion x-tra and Enamel Plus HRi Bio Function Enamel showed a reduction to moderate cytotoxicity rates, while G-aenial Flo X and Enamel Plus HRi Bio Function Bio Dentine showed similar results after 48 h and 72 h [39]. Kavuncu et al. investigated nanohybrid ormocerAdmira Fusion, Charisma Topaz, and resin-based EsteliteQuick Sigma [40]. Using MTT assay, they found that Admira Fusion and EsteliteQuick Sigma induced no cytotoxicity on human gingival fibroblasts after 24 h, while Charisma Topaz was cytotoxic [40]. Similarly, after one week of exposure, only Charisma Topaz was significantly cytotoxic. In the same study, for human periodontal ligament fibroblasts minor differences were observed in comparison to human gingival fibroblasts. Charisma Topaz was not significantly cytotoxic after 24 h when compared to the control group [40]. In one week, only Charisma Topaz was significantly cytotoxic, and cell viability was lower than in the control group [40]. For all materials, cytotoxicity was higher after one week than 24 h [40]. Sulek et al. investigated interactions of RBCs Charisma, Estelite Sigma Quick, and Filtek Z550 with human gingival fibroblasts. MTT assay revealed that all freshly cured materials caused a significant decrease in cell viability with the highest toxicity (a 58% decrease in viable cell numbers) induced by Charisma, while Estelite Sigma Quick and Filtek Z550 caused 22% and 31% declines, respectively [41]. Charisma was slightly less cytotoxic in the pre-cured application, while Filtek Z550 increased its toxicity considerably, and Estelite Sigma Quick induced similar toxicity in this state [41]. All three materials induced damage to the cell membrane. LDH assay used to assess time-dependent cytotoxicity saw increased LDH activity in cells incubated with Charisma after 1 h [41]. After 24 h, cell membrane damage increased to more than 40% of total enzyme activity, and the cytotoxicity plot was hyperbolic [41]. The trend of LDH release was similar for Estelite Sigma Quick, but the maximal values after 24 h were lower, reaching 28% of total enzyme activity [41]. Meanwhile, Filtek Z550 produced 37% of total enzyme activity after 24 h and, interestingly, the time-dependent cytotoxicity plot was a linear correlation [41].

### 4.3. Impact on Cell Cycle and Mechanism of Cell Death

The production of reactive oxygen species and depletion of cellular glutathione reserves described before is the likely reason for the induction of cell death in pulp cells, gingival fibroblasts, odontoblasts, and other cells exposed to monomers [25]. Evidence regarding the specific pathway of cell death induced by RBCs is scarce and inconclusive. This knowledge gap needs to be addressed, as it is relevant to the identification of efficient prevention strategies.

There are two pathways of cell death—apoptosis and necrosis. Cytotoxicity and genotoxicity via violation of DNA integrity are closely connected to apoptosis, which is a programmed cell death [47]. This pathway is regulated by proteolytic cysteinyl enzymes caspases. Caspase-3 acts as an executioner caspase which initiates DNA strand breaking and fragmentation [47,48,49]. This caspase can be activated via intrinsic or extrinsic pathways. Intrinsic pathway is connected to caspase-9 activity, which is triggered by mitochondrial disruption. The extrinsic pathway, meanwhile, is mediated by caspase-8 via death receptor activation [47]. The accumulation of intracellular reactive oxygen species is responsible for the activation of caspase chain [47,48].

Neves et al. found that incubation with BisGMA and *Porphyromonas gingivalis* resulted in a significant increase in the percentage of necrotic monocytes when compared with culture exposed to BisGMA alone [36]. As for TEGDMA, there was a noticeable increase in apoptotic cells in monocytes incubated with TEGDMA and *P. gingivalis* compared to exposure to *P. gingivalis* alone, while no such differences were observed for necrotic cells, thus suggesting that most of TEGDMA-induced cell death was via apoptosis [36]. UDMA was similarly found to induce cell death due to apoptosis [36]. Lovász et al. investigated the pathway of apoptotic cell death induced by TEGDMA in dental pulp cells [45]. They observed that TEGDMA exposure caused an increase in caspases and apoptosis-induced factor (AIF) production. Caspase-3 was significantly increased after exposure to 1.5 mM and 3 mM TEGDMA, caspase-8 in 0.1 and 0.2 mM, and caspase-9 in 0.75, 1.5 and 3 mM. Caspase-12 was significantly increased at concentrations above 0.75 mM. The production of AIF—a mitochondrial polypeptide responsible for chromatin condensation and DNA degradation—was induced by 0.2, 0.75, and 1.5 mM TEGDMA [45]. Similar induction of caspase-3, caspase-8, and caspase-9 activity in mouse macrophage after TEGDMA exposure at the concentration of 3 μM was found by Yang et al. [47]. These results indicate that TEGDMA-induced apoptosis, as evidenced by the increase in apoptosis-specific caspases. This information is of clinical relevance, as apoptosis does not involve an inflammatory process, in contrast to necrosis. There is no satisfactory evidence, however, to determine the specific pathway of apoptosis. Chang et al. studied the level of apoptosis and necrosis induced by UDMA in macrophages and proved that low concentrations induced early apoptosis, while at high concentrations, late apoptosis and necrosis were induced [49]. In another study by Wawrzynkiewicz et al. on human monocytes/macrophage peripheral blood cells, only OptiBond Universal induced a significant increase in apoptosis, as approximately 45% of cells were found at the early or late stage of apoptosis [51].

In the study by Sulek et al., the pathway of cell death in human gingival fibroblasts after 24 h incubation with RBCs was investigated using Annexin V/propidium iodide [41]. Estelite Sigma Quick produced an inconclusive pattern of changes with 48% necrotic cells and 28% of cells with mixed apoptotic and necrotic cells [41]. Filtek Z550 and Charisma produced mostly nonspecific necrotic changes, with 73% and 75% of cells stained as necrotic, respectively [41]. In cells incubated with Filtek Z550 a substantial number of cells—16%—developed both necrotic and apoptotic features, however. Wawrzynkiewicz et al. observed via FITC Annexin V apoptosis kit that G-Premio Bond induced a significant increase in the apoptosis of human peripheral blood cells, with approximately 39% of cells at the early or late stage of apoptosis [50].

Free monomers have been found to inhibit cell proliferation [25]. Sulek et al. demonstrated that Charisma, Estelite Sigma Quick, and Filtek Z550 caused significant antiproliferative effects [41]. Estelite Sigma Quick inhibited cell proliferation by about 60%, Filtek Z550 by 35%, and Charisma by 17% [41]. Interestingly, only Charisma additionally decreased the number of resting cells by 40% [41]. Sun et al. observed a time- and dose-dependent anti-proliferative influence of HEMA in human dental pulp cells [46]. HEMA was also found by Lee et al. to induce dose-dependent cytotoxicity via apoptosis and not necrosis in murine macrophages [48]. The intrinsic pathway of apoptosis was activated further in this study.

Mitochondrial dysfunction and toxic influence on DNA were suggested as possible mechanisms of apoptosis induction [36]. TEGDMA-induced apoptosis has been explained by the generation of reactive oxygen species, phosphorylation of mitogen-activated protein kinase, and downstream transcription factors [47].

### 4.4. Prevention Strategies

Prevention strategies are of vital importance in clinical settings as RBCs remain widespread. Selecting the most biocompatible monomers is an important direction in the development of more suitable composite resins. This approach has its limitations, however, as other factors, such as the degree of conversion or the species of photo-initiator, can influence cytotoxicity, accounting for differences in cytotoxicity levels in materials with the same monomer composition. The quantity and composition of eluate should both be taken into consideration separately, as there is no direct relation between the amount of released monomers and the composition of the biomaterial [30]. For example, while many RBCs contain a mixture of BisGMA and TEGDMA, the more hydrophilic TEGDMA is likely to be eluted in higher quantities [30].

Dentin has been reported to have a physiological protective effect [30]. Carrillo-Cotto et al. tried to replicate the conditions of the oral cavity by investigating the cytotoxicity of RBCs on keratinocytes in the presence of dentin and without [38]. Interestingly, they found out that after 1 day of incubation, the cell viability of the control group was ten times higher in the presence of dentin. This behavior was observed for all groups where dentin was present [38]. Dentin mechanically lowers the concentration of toxic substances, serving as a diffusion barrier [30]. It is also possible for dentin to absorb the unbound monomers and exert a buffering effect [30,38]. Dentin was observed to stimulate proliferation and increase cell viability. The responsible mechanism may be explained by the presence of growth factors in this tissue and its release into the medium [38]. Based on these findings, it should be investigated whether the presence of growth factors in dentin is capable of counteracting the cytotoxic impact of leached monomers from RBCs in vivo. Indirect contact tests with dentin should be used to produce an understanding of hard tissue interactions with diffusing monomers [11].

Antioxidants such as rutin or melatonin have been proposed with some success as a prevention method to reduce the genotoxicity of RBCs [25,47]. Cengiz et al. investigated the possible protective influence of melatonin in saliva, as melatonin is reported to have properties such as DNA protection, reducing inflammation, antioxidative characteristics, and potentially being an antiapoptotic agent [37,56]. However, the MTT assay revealed no significant differences in cytotoxicity between murine fibroblasts incubated in artificial saliva with the addition of melatonin and those without [37]. Yang et al. investigated the protective effect of rutin—a bioflavonoid antioxidant—on TEGDMA-induced cytotoxicity in murine macrophages [47]. Rutin, also known as quercetin-3-rhamnosyl glucoside, is a natural flavanol glycoside known to reduce inflammatory response and genotoxicity [47]. Pretreatment with rutin was found to decrease TEGDMA cytotoxicity in a concentration-dependent manner, with significant reduction beginning at 30 μM [47]. Genotoxicity, apoptosis, necrosis, and reduction in reactive oxygen species generation decreased similarly in the same study.

Another important factor determining the extent of cytotoxicity is the degree of polymerization, measured as the Degree of Conversion, which is dependent on curing time, light source, viscosity and thickness of the RBCs layer, type and mixture of photo-initiators as well as type and proportion of monomers and filler [11,39,40]. Incomplete polymerization contributes to the increased release of unreacted monomers and therefore supports cytotoxicity [11,12,38,39,40,43]. The monomer composition of the material is not the only factor influencing its biocompatibility. Filler content should also be considered. It has been found that nano-hybrid ormocers exert less cytotoxicity and release less unbound monomers [12,39,40]. A higher filler content minimizes the organic resin component, thus improving biocompatibility [39]. Beltrami et al. have found that Omnichroma, Omnichroma Blocker, Admira Fusion x-tra, and Enamel Plus HRi Bio Function Enamel—in all of which classic monomers are absent—induced lower cytotoxicity in human gingival fibroblasts when compared with conventional materials. Similarly, Kavuncu et al. investigated nanohybrid ormocerAdmira Fusion, nanohybrid Charisma Topaz, and resin-based Estelite Quick Sigma in human gingival fibroblasts and periodontal ligament fibroblasts. Their findings indicated Charisma Topaz as the most toxic material of the three after both 24 h and 1 week exposure [40]. This suggests that ormocer group composites can be considered the most biocompatible materials in clinical cases, especially in close contact with gingiva and periodontium.

Pulp capping refers to the process of placing capping material, e.g., calcium hydroxide, over the exposed pulp in order to preserve its vitality and protect against toxic substances and physical stimuli. Novel capping materials include mineral trioxide aggregate (MTA). Restorative material is then placed directly over the capping material. RBCs are known to impair healing processes and the formation of a reparative dentin barrier while in direct contact with the exposed pulp [30]. Agnes et al. investigated possible combined toxic effects of RBCs and capping materials in human dental pulp cells [44]. They found that calcium hydroxide Dycal demonstrated dose-dependent toxicity, while MTA remained bioinert. With cultures exposed to Dycal and RBCs Durafill or Flow Line for 24 h, cell death rates reached 30–40%. Meanwhile, MTA increased the cytotoxicity of Durafill but had no impact on the Flow Line. Flow Line and Durafill were also found to induce some oxidative stress, but the results varied between groups. Interestingly, MTA enhanced oxidative stress induced by Durafill while Dycal reduced the oxidative stress of Flow Line. This study suggests the need for further investigations into the interactions between pulp capping materials and restorative composites. Another issue in pulp capping is to research the ability of eluted monomers able to diffuse through the capping material.

### 4.5. Analysis of Methodologies

RBCs should undergo several steps of biocompatibility and toxicity tests. The more standardized, systematic approach toward methodology should be developed to enable qualitative and quantitative analysis and synthesis of results with clinical relevance. Presently, however, there is little cohesion in methodologies among studies in the field [11]. Methodological standardization should be of primary concern to provide easier comparison of studies and reduce conflicting results.

The oral cavity is a complex environment with many diverse niches and interactions between host, restorative material, and microorganisms that are hard to replicate in in vitro studies. Limited volume, flow of saliva and dentinal fluid, and chemical interactions with dentine are some of the factors that contribute to the complexity of replicating this environment.

In vitro cytotoxicity tests are the first step in the evaluation of biocompatibility. In biocompatibility studies, there are three modes of in vitro methods, as outlined in Table 5. Direct contact tests are the least complex and therefore widely used. However, scientists argue that direct contact tests are actually of limited clinical relevance, as most cells are not in direct contact with the biomaterial on site [25]. As such, an indirect contact test seems to be a preferable alternative. Of these methods, an indirect contact test with a dentin layer can yield clinically relevant results when testing RBCs’ impact on the pulp tissue.

Many studies on the subject tend to report the influence of resin composite materials at TC_50_ (concentration that caused a 50% decrease in cell viability) concentrations. However, some authors argue that such high concentrations of eluted monomers are not likely to be released from fillings in the site. As such, a lower concentration of methacrylates, such as TC_20_, could yield more relevant results [36]. The determination of TC_50_ concentration is also dependent on the type of host cells investigated [36]. This is contested by other researchers who argue that limited volume, especially in niches such as the pulp chamber, can allow for the accumulation of higher concentrations of eluted monomers [42]. Monomers such as TEGDMA and HEMA can diffuse through dentinal tubules, reaching the pulp chamber in toxic concentrations [11]. The highest intrapulpal concentration has been reported as 4 mM [43]. The quantity of monomers released from polymerized RBCs is dependent on the ratio of the material sample surface to cell culture volume [30,39,40]. In clinical practice, the mean surface area of mesial-occlusal-distal fillings was calculated as 95 mm^2^, in cervical fillings as 12 mm^2^, and in veneers as 86 mm^2^ [40].

In biocompatibility studies, some continuous cell lines, e.g., mouse fibroblasts, are commonly used due to their easy production and control of the culture. However, it should be noted that using the primary cells of the target tissue, e.g., pulp cells and gingival or periodontal ligament fibroblasts, results in studies of higher quality, producing more meaningful outcomes to consider in clinical applications [11]. Thus, primary cells should be preferred, regardless of the difficulties they might cause in the laboratory, e.g., slower growth or shorter life span of the cells. It is important to remember that monolayer cell cultures exhibit higher sensitivity to toxins than three-dimensional (3D) cultures, which is an important limitation of most in vitro studies. Substitution of monolayer cell culture with micro-tissue 3D models allows improved replication of the tissue microenvironment by enabling cellular communication and increased cell–cell and extracellular matrix–cell interactions [11,30].

Longer experimental periods could provide further insight into the chronic effects of the continuous long-term elution of unbound monomers on cell cultures, as such chronic exposure should not be discarded in human health risk considerations [39,40].

Interpretation of the underlying molecular mechanisms of toxicity is complicated, and it is hard to establish causality—biochemical changes are measured after toxicity has already occurred and may be either the cause of the cell death or its result. Appropriate selection of viability assay is another important consideration. The colorimetric MTT assay is widely described in the literature. It delivers objective results in a short time and is based on the assessment of cell metabolic activity. MTT is also recommended by international standards, including ISO 10993 [11]. However, its results can be affected by cell numbers and provide evidence of cell death only, bringing no contribution to the understanding of cytotoxicity pathways [11,41]. LDH release assay is frequently used as well and delivers information on the measure of dead cells. Toxicologists have established new paradigms that go beyond the measure of cell death and deliver additional information on the mechanism of toxicity based on the methodologies of proteomics, genomics, and pathway analyses, but these paradigms are only beginning to influence dental materials research [11,30]. Such methods include scanning electron microscopy to observe cell morphology, investigation of necrosis and apoptosis pathways, and flow cytometry to evaluate the effects on cell cycle [11]. Without a comprehensive understanding of the mechanisms of toxicity, materials with improved biocompatibility cannot be developed.

## 5. Summary

In this manuscript, the authors analyzed in what way resin-based composite materials used in dental practice induce cytotoxicity in the oral cavity tissues. Biocompatibility of dental materials remains an important factor in the development of novel RBCs. In recent years, there has been significant development in the field. RBCs have been markedly improved in regard to their aesthetic value, adhesive abilities, and mechanical properties. Researchers and manufacturers strive to further improve these materials, focusing on reducing their polymerization shrinkage, preventing secondary caries, strengthening adhesion, and further improving durability and resistance to degradation or endowing materials with bioactive, antibacterial properties. Safety profile considerations should be taken into account in the early stages of these developments. Analyzed papers point out that most resin-based composites induce dose- and time-dependent cytotoxic effects, causing oxidative stress and depletion of cellular glutathione reserves as well as disrupting enzymatic activity. Interestingly, they were also found in several studies to induce apoptosis. Viability loss, oxidative stress, depletion of glutathione, and induction of apoptosis in cells exposed to composite materials is well documented, and other studies support the conclusions of this manuscript [57]. The results of this review indicate that there is still a need for further research and improvement in this area. According to the current state of knowledge, RBCs meet legal regulations and standards, such as the ones outlined in ISO 14971, ISO 10993, and ISO 7405. However, the acquisition of knowledge concerning biocompatibility can provide a better understanding of material–host interaction and lead to the development of novel materials with improved safety profiles or the introduction of clearer safety measures in clinical practice. The benefits of such developments for the patients, clinicians, public and environmental health are obviously overwhelming, even if hard to quantify. Possible solutions which can improve the biocompatibility of dental resin-based composite materials include improving their degree of conversion and resistance to weardown, thus reducing the number of free monomers. Monomers liberated from these materials should be carefully identified and quantified as they determine the extent of cytotoxicity. Research indicates that newer monomers tend to present higher biocompatibility than older types, such as BisGMA. Direct and indirect pulp capping is recommended to protect the pulp from exposure and enable reparative dentin formation. The addition of antioxidants is being considered to reduce oxidative stress. Nanomaterials are reported to present great improvement in material properties, including biocompatibility [30]. There is also a need for the development of more standardized methods of in vitro tests for use in dental material science, which will allow for more accurate conclusions and comparisons and eliminate conflicting outcomes. Biocompatibility research in this area should provide more insight into the molecular mechanisms of cytotoxicity, which are not yet fully understood. A better understanding of these mechanisms may drive forward the development of more biocompatible materials. It should be noted that in vitro settings are limited in their ability to represent the intricately complex and changing environment of the oral cavity. The components of saliva and dentinal fluid, the presence of dentin and the quality of monomers capable of diffusing through this tissue to the pulp, the impact of physiological and pathological bacteria, changes in pH, and mechanical factors all influence the release of resin monomers from composite restorations and, as such, have impact on the adverse effects of resin-based composite materials in the patient.

## Figures and Tables

**Table 1:** Function and composition of resin-based composite phases based on [5,10,27,28].

Phase	Function	Composition
Resin matrix	Polymerization	BisGMA, TEGDMA, UDMA, HEMA, BisEMA, EGDMA
Filler particles	Improvement in mechanical and wear properties, aesthetic qualities, and reduction in polymerization shrinkage	Soft and hard glass: borosilicate, quartz, aluminum silicate, lithium aluminum silicate, ytterbium fluoride, barium, strontium, zirconium and zinc glass
Coupling agent	Combining resin matrix and filler particles, reduced water sorption	Silanes, zirconates, titanates
Photo-initiator	Initiation of resin matrix polymerization by providing free radicals upon exposure to external energy	CQ, PQ, TPO

BisGMA—bisphenol A-glycidyl methacrylate; TEGDMA—triethylene glycol dimethacrylate; UDMA—urethane dimethacrylate; HEMA—2-hydroxyethyl methacrylate; BisEMA—ethoxylatedbisphenol-A dimethacrylate; EGDMA—ethylene glycol dimethacrylate; CQ—camphorquinone; PQ—phenanthrenequinone; TPO—trimethylbenzoyl-diphenylphosphine oxide.

**Table 2:** The inclusion and exclusion criteria.

Inclusion Criteria	Exclusion Criteria
Publication in English Published since 2017 In vitro study Containing the following keyword combinations: “cytotoxicity of dental materials” OR “cytotoxicity of resin composite” OR “biocompatibility of dental materials” OR “biocompatibility of resin composite” OR “oxidative stress of dental materials” OR “oxidative stress of resin composite” OR “genotoxicity of dental materials” OR “genotoxicity of resin composite” OR “mutagenicity of dental materials” OR “mutagenicity of resin composite”	Publication not in English Published before 2017 Review articles, abstracts, book chapters, live animal studies

**Table 3:** The Quality Assessment Tool for In Vitro Studies (the QUIN Tool) risk of bias.

Study	Final Score (%)	Risk of Bias
Neves et al., 2019 [36]	65	Medium
Cengiz et al., 2022 [37]	80	Low
Carrillo-Cotto et al., 2020 [38]	80	Low
Beltrami et al., 2021 [39]	70	Medium
Kavuncu et al., 2020 [40]	70	Medium
Sulek et al., 2022 [41]	80	Low
Schneider et al., 2019 [42]	55	Medium
Lovász et al., 2021 [43]	55	Medium
Agnes et al., 2017 [44]	80	Low
Lovász et al., 2021 [45]	80	Low
Sun et al., 2018 [46]	75	Low
Yang et al., 2022 [47]	85	Low
Lee et al., 2022 [48]	75	Low
Chang et al., 2020 [49]	75	Low
Wawrzynkiewicz et al., 2021 [50]	85	Low
Wawrzynkiewicz et al., 2020 [51]	85	Low

**Table 4:** Summary of study characteristics.

Study	Cell Viability Assay	Other Methods	Cell Line	Resin-Based Materials
Neves et al., 2019 [36]	MTT assay	APC Annexin V apoptosis detection kit, ELISA assay	Human peripheral blood mononuclear cells (hPBMC)	Monomers: BisGMA, TEGDMA, UDMA
Schneider et al., 2019 [42]	LDH assay	MCB assay, DCF assay	Human dental pulp cells (hDPC)	Monomers: BisGMA, TEGDMA, UDMA
Lovász et al., 2021 [43]	WST-1 assay	EncCheckGelatinolyticCollagenolitic activity assay, Western blotting	Human dental pulp cells (hDPC)	Monomers: TEGDMA
Lovász et al., 2021 [45]	WST-1 assay	Hemocytometer, fluorescence microscopy, Western blotting	Human dental pulp cells (hDPC)	Monomers: TEGDMA
Sun et al., 2018 [46]	MTT assay	RT-PCR, gelatinezymography, transwell migration assay, Western blotting	Human dental pulp cells (hDPC)	Monomers: HEMA
Yang et al., 2022 [47]	MTT assay	FITC Annexin V, comet assay	Murine macrophages	Monomers: TEGDMA
Lee et al., 2022 [48]	MTT assay	FITC Annexin V, micronucleus assay, comet assay	Murine macrophages	Monomers: HEMA
Chang et al., 2020 [49]	LDH assay	FITC Annexin V, MN assay, comet assay	Murine macrophage	Monomers: UDMA
Cengiz et al., 2022 [37]	MTT assay	None	Murine fibroblasts (mF)	Eluates of polymerized specimens: Signum (S), Adoro (A)
Carillo-Cotto et al., 2020 [38]	MTT assay	Fourier-transform infrared spectroscopy	Human keratinocytes (hK)	Eluates of polymerized specimens: OptiBond FL (OB), Clearfil SE Bond (CB), Adper Single Bond Universal (AS), Filtek Z350 XT (FZ3), Filtek Flow Z350 XT (FFZ3), Dyad Flow (DF), Variolink II (VII), RelyXU200 (RX)
Beltrami et al., 2021 [39]	MTT assay	None	Human gingival fibroblasts (hGF)	Eluates of polymerized specimens: Omnichroma (OC), Omnichroma Blocker (OCB), Admira Fusion x-tra (AFX), Enamel Plus Hri Bio Function Enamel (EPE), Enamel Plus Hri (EP), G-aenial (GA), G-aenial Flo X (GAF), Enamel Plus Hri Bio Function Bio Dentine (EPD)
Kavuncu et al., 2020 [40]	MTT assay	None	Human gingival fibroblasts (hGF), human periodontal ligament fibroblasts (hPLF)	Polymerized samples placed directly in cell culture medium: Admira Fusion (AF), Charisma Topaz (CT), Estelite Sigma Quick (ESQ)
Sulek et al., 2022 [41]	MTT assay, LDH assay	Flow cytometry, FITC Annexin V, Western blotting	Human gingival fibroblasts (hGF)	Eluates of polymerized specimens: Charisma (CH), Estelite Sigma Quick (ESQ), Filtek Z550 (FZ5)
Agnes et al., 2017 [44]	LDH assay	DCF assay	Human dental pulp cells (hDPC)	Polymerized samples placed directly in cell culture medium: Flow Line (FL), Durafill VS (DF)
Wawrzynkiewicz et al., 2021 [50]	XTT assay	Comet assay, flow cytometry, FITC Annexin V	Human monocytes/macrophage peripheral blood cells	Eluates of polymerized specimens: All-Bond Universal, CLEARFIL Universal Bond Quick, G-Premio BOND, Single Bond Universal
Wawrzynkiewicz et al., [51]	XTT assay	Comet assay, flow cytometry, FITC Annexin V	Human monocytes/macrophage peripheral blood cells	Eluates of polymerized specimens: OptiBond Universal, Prime&Bond Universal, AdheseUniversal

MTT—3-(4,5-dimethylthiazol-2-yl)-2,5-diphenyl-2H-tetrazolium bromide; LDH—L-lactate dehydrogenase; WST-1—2-(4-iodophenyl)-3-(4-nitrophenyl)-5-(2,4-disulfophenyl)-2H-tetrazolium; XTT—2,3-bis (2-methoxy-4-nitro-5-sulfophenyl)-5-[(phenylamino) carbonyl]-2H-tetrazolium hydroxide; BisGMA—bisphenol A-glycidyl methacrylate; TEGDMA—triethylene glycol dimethacrylate; UDMA—urethane dimethacrylate; HEMA—2-hydroxyethyl methacrylate.

**Table 5:** In vitro methods in biocompatibility studies [12,25,40].

Method	Characteristics
Direct contact test	Direct contact between the material and cell culture, typically in mono-layer.
Indirect contact test	Separation of the material and cell culture with an intermediate layer, e.g., agar gel, Millipore filter, dentin layer.
Extract test	Application of eluates from the material to cell culture.

## References

[1] Bin-Jardan L.I., Almadani D.I., Almutairi L.S., Almoabid H.A., Alessa M.A., Almulhim K.S., AlSheikh R.N., Al-Dulaijan Y.A., Ibrahim M.S., Al-Zain A.O. (2023). Inorganic Compounds as Remineralizing Fillers in Dental Restorative Materials: Narrative Review. Int. J. Mol. Sci..

[2] Al-hijazi A.Y., Hasan N., Nasr B.K., Jasim Al-Khafaji H.H., Al-Khafaji B., Abdah Alanssari B.F., Jalil A.T. (2023). Recent Advances in the Use of Inorganic Nanomaterials as Anti Caries Agents. Heliyon.

[3] Guo X., Yu Y., Gao S., Zhang Z., Zhao H. (2022). Biodegradation of Dental Resin-Based Composite—A Potential Factor Affecting the Bonding Effect: A Narrative Review. Biomedicines.

[4] Berghaus E., Klocke T., Maletz R., Petersen S. (2023). Degree of Conversion and Residual Monomer Elution of 3D-Printed, Milled and Self-Cured Resin-Based Composite Materials for Temporary Dental Crowns and Bridges. J. Mater. Sci. Mater. Med..

[5] Riva Y.R., Rahman S.F. (2019). Dental Composite Resin: A Review. AIP Conf. Proc..

[6] Demarco F.F., Cenci M.S., Montagner A.F., De Lima V.P., Correa M.B., Moraes R.R., Opdam N.J.M. (2023). Longevity of Composite Restorations Is Definitely Not Only about Materials. Dent. Mater..

[7] Gitalis R., Zhou L., Marashdeh M.Q., Sun C., Glogauer M., Finer Y. (2019). Human Neutrophils Degrade Methacrylate Resin Composites and Tooth Dentin. Acta Biomater..

[8] Smith L., Ali M., Agrissais M., Mulligan S., Koh L., Martin N. (2023). A Comparative Life Cycle Assessment of Dental Restorative Materials. Dent. Mater..

[9] Arbildo-Vega H.I., Lapinska B., Panda S., Khan A.S., Lukomska-Szymanska M. (2020). Clinical Effectiveness of Bulk-Fill and Conventional Resin Composite Restorations: Systematic Review and Meta-Analysis. Polymers.

[10] Pratap B., Gupta R.K., Bhardwaj B., Nag M. (2019). Resin Based Restorative Dental Materials: Characteristics and Future Perspectives. Jpn. Dent. Sci. Rev..

[11] Caldas I.P., Alves G.G., Barbosa I.B., Scelza P., de Noronha F., Scelza M.Z. (2019). In Vitro Cytotoxicity of Dental Adhesives: A Systematic Review. Dent. Mater..

[12] Bapat R.A., Parolia A., Chaubal T., Dharamadhikari S., Abdulla A.M., Sakkir N., Arora S., Bapat P., Sindi A.M., Kesharwani P. (2021). Recent Update on Potential Cytotoxicity, Biocompatibility and Preventive Measures of Biomaterials Used in Dentistry. Biomater. Sci..

[13] Wuersching S.N., Högg C., Kohl L., Reichl F.-X., Hickel R., Kollmuss M. (2023). Leaching Components and Initial Biocompatibility of Novel Bioactive Restorative Materials. Dent. Mater..

[14] Lankes V., Reymus M., Mayinger F., Coldea A., Liebermann A., Hoffmann M., Stawarczyk B. (2023). Three-Dimensional Printed Resin: Impact of Different Cleaning Protocols on Degree of Conversion and Tensile Bond Strength to a Composite Resin Using Various Adhesive Systems. Materials.

[15] Jung Y.S., Ro S.T., Kang S.W., Lee H., Lee J.S., Chae Y.K., Lee K.E., Lee H.-S., Kwack K.H., Kim S.K. (2023). Bisphenol A Release from Commercially Available 3-Dimensionally Printed Resins and Human Cell Apoptosis to Bisphenol A: An in-Vitro Study. J. Clin. Pediatr. Dent..

[16] Vulović S., Nikolić-Jakoba N., Radunović M., Petrović S., Popovac A., Todorović M., Milić-Lemić A. (2023). Biofilm Formation on the Surfaces of CAD/CAM Dental Polymers. Polymers.

[17] Tiu J., Belli R., Lohbauer U. (2023). A Step toward Bio-Inspired Dental Composites. Biomater. Investig. Dent..

[18] Khan A.A., Zafar M.S., Fareed M.A., AlMufareh N.A., Alshehri F., AlSunbul H., Lassila L., Garoushi S., Vallittu P.K. (2023). Fiber-Reinforced Composites in Dentistry—An Insight into Adhesion Aspects of the Material and the Restored Tooth Construct. Dent. Mater..

[19] He J., Lassila L., Garoushi S., Vallittu P. (2023). Tailoring the Monomers to Overcome the Shortcomings of Current Dental Resin Composites—Review. Biomater. Investig. Dent..

[20] Hardan L., Devoto W., Bourgi R., Cuevas-Suárez C.E., Lukomska-Szymanska M., Fernández-Barrera M.Á., Cornejo-Ríos E., Monteiro P., Zarow M., Jakubowicz N. (2022). Immediate Dentin Sealing for Adhesive Cementation of Indirect Restorations: A Systematic Review and Meta-Analysis. Gels.

[21] Frassetto A., Breschi L., Turco G., Marchesi G., Di Lenarda R., Tay F.R., Pashley D.H., Cadenaro M. (2016). Mechanisms of Degradation of the Hybrid Layer in Adhesive Dentistry and Therapeutic Agents to Improve Bond Durability—A Literature Review. Dent. Mater..

[22] Zhang X., Zhang Q., Meng X., Ye Y., Feng D., Xue J., Wang H., Huang H., Wang M., Wang J. (2021). Rheological and Mechanical Properties of Resin-Based Materials Applied in Dental Restorations. Polymers.

[23] Cervino G., Cicciù M., Herford A.S., Germanà A., Fiorillo L. (2020). Biological and Chemo-Physical Features of Denture Resins. Materials.

[24] Mulligan S., Hatton P.V., Martin N. (2022). Resin-Based Composite Materials: Elution and Pollution. Br. Dent. J..

[25] Shahi S., Özcan M., Maleki Dizaj S., Sharifi S., Al-Haj Husain N., Eftekhari A., Ahmadian E. (2019). A Review on Potential Toxicity of Dental Material and Screening Their Biocompatibility. Toxicol. Mech. Methods.

[26] Pałka K., Miazga-Karska M., Pawłat J., Kleczewska J., Przekora A. (2021). The Effect of Liquid Rubber Addition on the Physicochemical Properties, Cytotoxicity, and Ability to Inhibit Biofilm Formation of Dental Composites. Materials.

[27] Szczesio-Wlodarczyk A., Domarecka M., Kopacz K., Sokolowski J., Bociong K. (2021). An Evaluation of the Properties of Urethane Dimethacrylate-Based Dental Resins. Materials.

[28] Kowalska A., Sokolowski J., Bociong K. (2021). The Photoinitiators Used in Resin Based Dental Composite—A Review and Future Perspectives. Polymers.

[29] Mulla S.A., Kondkari S.A., Patil A., Jain A., Mali S., Jaiswal H.C., Jakhar A., Ansari Z.M., Agarwal S., Yadav P. (2023). A Look Into the Cytotoxicity of Composite Fillings: Friend or Foe?. Cureus.

[30] Schmalz G., Galler K.M. (2017). Biocompatibility of Biomaterials—Lessons Learned and Considerations for the Design of Novel Materials. Dent. Mater..

[31] Arab-Nozari M., Zamani E., Evazalipour M., Soleimani B., Jorbonian A., Nahvi A. (2023). Histocompatibility of Light-Curing Composites Used in Pediatric Dentistry in Human Oral Fibroblast Cells. J. Dent..

[32] Folwaczny M., Ahantab R., Kessler A., Ern C., Frasheri I. (2023). Cytotoxicity of 3D Printed Resin Materials for Temporary Restorations on Human Periodontal Ligament (PDL-hTERT) Cells. Dent. Mater..

[33] Zhang K., Zhang N., Weir M.D., Reynolds M.A., Bai Y., Xu H.H.K. (2017). Bioactive Dental Composites and Bonding Agents Having Remineralizing and Antibacterial Characteristics. Dent. Clin. N. Am..

[34] Sheth V.H., Shah N.P., Jain R., Bhanushali N., Bhatnagar V. (2022). Development and Validation of a Risk-of-Bias Tool for Assessing in Vitro Studies Conducted in Dentistry: The QUIN. J. Prosthet. Dent..

[35] Page M.J., McKenzie J.E., Bossuyt P.M., Boutron I., Hoffmann T.C., Mulrow C.D., Shamseer L., Tetzlaff J.M., Akl E.A., Brennan S.E. (2021). The PRISMA 2020 Statement: An Updated Guideline for Reporting Systematic Reviews. BMJ.

[36] Neves S.O., Magalhães L.M.D., Corrêa J.D., Dutra W.O., Gollob K.J., Silva T.A., Horta M.C.R., Souza P.E.A. (2019). Composite-Derived Monomers Affect Cell Viability and Cytokine Expression in Human Leukocytes Stimulated with Porphyromonas Gingivalis. J. Appl. Oral. Sci..

[37] Cengiz S., Velioğlu N., Cengiz M.İ., Çakmak Özlü F., Akbal A.U., Çoban A.Y., Özcan M. (2022). Cytotoxicity of Acrylic Resins, Particulate Filler Composite Resin and Thermoplastic Material in Artificial Saliva with and without Melatonin. Materials.

[38] Carrillo-Cotto R., Etges A., Jardim P.S., Torre E., Kaizer M.R., Ferrúa C.P., Nedel F., Cuevas-Suárez C.E., Moraes R.R. (2020). Cytotoxicity of Contemporary Resin-based Dental Materials in Contact with Dentin. Eur. J. Oral Sci..

[39] Beltrami R., Colombo M., Rizzo K., Di Cristofaro A., Poggio C., Pietrocola G. (2021). Cytotoxicity of Different Composite Resins on Human Gingival Fibroblast Cell Lines. Biomimetics.

[40] Kavuncu G., Yilmaz A.M., Karademir Yilmaz B., Yilmaz Atali P., Altunok E.C., Kuru L., Agrali O.B. (2020). Cytotoxicity of Different Nano Composite Resins on Human Gingival and Periodontal Ligament Fibroblast Cell Lines: An In Vitro Study. Biomedicines.

[41] Sulek J., Luczaj-Cepowicz E., Marczuk-Kolada G., Rosłan M., Holownia A. (2022). Cytotoxicity of Methacrylate Dental Resins to Human Gingival Fibroblasts. J. Funct. Biomater..

[42] Schneider T.R., Hakami-Tafreshi R., Tomasino-Perez A., Tayebi L., Lobner D. (2019). Effects of Dental Composite Resin Monomers on Dental Pulp Cells. Dent. Mater. J..

[43] Lovász B.V., Lempel E., Szalma J., Sétáló G., Vecsernyés M., Berta G. (2021). Influence of TEGDMA Monomer on MMP-2, MMP-8, and MMP-9 Production and Collagenase Activity in Pulp Cells. Clin. Oral Investig..

[44] Agnes A., Long A., Best S., Lobner D. (2017). Pulp Capping Materials Alter the Toxicity and Oxidative Stress Induced by Composite Resins in Dental Pulp Culture. Eur. Endod. J..

[45] Lovász B.V., Berta G., Lempel E., Sétáló G., Vecsernyés M., Szalma J. (2021). TEGDMA (Triethylene Glycol Dimethacrylate) Induces Both Caspase-Dependent and Caspase-Independent Apoptotic Pathways in Pulp Cells. Polymers.

[46] Sun S., Wang G.-L., Huang Y., Diwu H.-L., Luo Y.-C., Su J., Xiao Y.-H. (2018). The Effects of 2-Hydroxyethyl Methacrylate on Matrix Metalloproteinases 2 and 9 in Human Pulp Cells and Odontoblast-like Cells in Vitro. Int. Endod. J..

[47] Yang L.-C., Chang Y.-C., Yeh K.-L., Huang F.-M., Su N.-Y., Kuan Y.-H. (2022). Protective Effect of Rutin on Triethylene Glycol Dimethacrylate-Induced Toxicity through the Inhibition of Caspase Activation and Reactive Oxygen Species Generation in Macrophages. Int. J. Mol. Sci..

[48] Lee C.-Y., Ho Y.-C., Lee S.-S., Li Y.-C., Lai M.-Y., Kuan Y.-H. (2022). Cytotoxicity and Apoptotic Mechanism of 2-Hydroxyethyl Methacrylate via Genotoxicity and the Mitochondrial-Dependent Intrinsic Caspase Pathway and Intracellular Reactive Oxygen Species Accumulation in Macrophages. Polymers.

[49] Chang C.-Y., Chiang C.-Y., Chiang Y.-W., Lee M.-W., Lee C.-Y., Chen H.-Y., Lin H.-W., Kuan Y.-H. (2020). Toxic Effects of Urethane Dimethacrylate on Macrophages Through Caspase Activation, Mitochondrial Dysfunction, and Reactive Oxygen Species Generation. Polymers.

[50] Wawrzynkiewicz A., Rozpedek-Kaminska W., Galita G., Lukomska-Szymanska M., Lapinska B., Sokolowski J., Majsterek I. (2021). The Toxicity of Universal Dental Adhesives: An In Vitro Study. Polymers.

[51] Wawrzynkiewicz A., Rozpedek-Kaminska W., Galita G., Lukomska-Szymanska M., Lapinska B., Sokolowski J., Majsterek I. (2020). The Cytotoxicity and Genotoxicity of Three Dental Universal Adhesives—An In Vitro Study. Int. J. Mol. Sci..

[52] Ghasemi M., Turnbull T., Sebastian S., Kempson I. (2021). The MTT Assay: Utility, Limitations, Pitfalls, and Interpretation in Bulk and Single-Cell Analysis. Int. J. Mol. Sci..

[53] Kuhn D.M., Balkis M., Chandra J., Mukherjee P.K., Ghannoum M.A. (2003). Uses and Limitations of the XTT Assay in Studies of *Candida* Growth and Metabolism. J. Clin. Microbiol..

[54] Scarcello E., Lambremont A., Vanbever R., Jacques P.J., Lison D. (2020). Mind Your Assays: Misleading Cytotoxicity with the WST-1 Assay in the Presence of Manganese. PLoS ONE.

[55] Kaja S., Payne A.J., Naumchuk Y., Koulen P. (2017). Quantification of Lactate Dehydrogenase for Cell Viability Testing Using Cell Lines and Primary Cultured Astrocytes. Curr. Protoc. Toxicol..

[56] Korkmaz A., Reiter R.J., Topal T., Manchester L.C., Oter S., Tan D.-X. (2009). Melatonin: An Established Antioxidant Worthy of Use in Clinical Trials. Mol. Med..

[57] Samuelsen J.T., Dahl J.E. (2023). Biological Aspects of Modern Dental Composites. Biomater. Investig. Dent..

